# Panophthalmitis Caused by Providencia stuartii: A Rare Opportunistic Ocular Infection

**DOI:** 10.7759/cureus.112240

**Published:** 2026-07-07

**Authors:** Preshaantini Ponnaiah, Chandramalar T. Santhirathelagan, Shamala Retnasabapathy, Wan Haslina Wan Abdul Halim

**Affiliations:** 1 Ophthalmology, Hospital Sungai Buloh, Universiti Kebangsaan Malaysia, Sungai Buloh, MYS; 2 Ophthalmology, Hospital Sungai Buloh, Sungai Buloh, MYS; 3 Ophthalmology, Universiti Kebangsaan Malaysia Medical Centre, Kuala Lumpur, MYS

**Keywords:** antibiotics, ocular infection, opportunistic infection, panophthalmitis, providencia stuartii

## Abstract

This case report describes a case of* Providencia* *stuartii* panophthalmitis, a rare opportunistic ocular infection. A 58-year-old lady with diabetes mellitus presented with painful vision loss in the left eye for one day. Visual acuity was light perception with a relative afferent pupillary defect. Examination showed limitation of extra-ocular muscle movement in all gazes, proptosis, erythematous lid swelling, conjunctival injection, oedematous cornea, and hypopyon with fibrin in the anterior chamber. B-scan showed vitritis, loculations, and scleritis. Vitreous culture revealed *P. stuartii. *Concurrently,contrast-enhanced computed tomography (CECT) of the orbit demonstrated left eye proptosis, circumferential scleral thickening and enhancement, choroidal enhancement, and fat stranding across both the intraconal and extraconal spaces, highly suggestive of panophthalmitis. She was treated with intravitreal vancomycin and ceftazidime, topical ciprofloxacin and ceftazidime, and systemic ciprofloxacin and meropenem. Due to corneal perforation, evisceration was done. Vitreous, uveal, and conjunctival cultures grew *P. stuartii*. Chemosis and eye discharge were reduced with topical cefuroxime and chloramphenicol, but with residual necrotic tissue. *P. *s*tuartii* infection is a rare and serious ocular infection with a poor visual outcome.

## Introduction

Endophthalmitis is a severe infection and inflammation of the intraocular cavity and its contents. Panophthalmitis is a more severe, rapidly progressing ocular infection and inflammatory process with devastating outcomes as it is sight- and globe-threatening. It involves all layers of the globe, orbit, and the periorbital structures [1,2].

Similar to endophthalmitis, panophthalmitis can be classified into exogenous or endogenous types depending on the source of infection. The common exogenous panophthalmitis is caused by an infective organism invading from the external environment following any ocular surgery or trauma. Panophthalmitis may arise from haematogenous spread of infection, commonly from systemic foci, such as liver abscess, lung infection, endocarditis, soft tissue infection, urinary tract infection, meningitis, septic arthritis, and orbital cellulitis [1,2].

In panophthalmitis and endophthalmitis, patients present with similar symptoms and signs, which include reduced vision, eye pain and redness, presence of hypopyon, and hazy fundus view due to the ongoing severe intraocular inflammation. In addition, proptosis, restriction of extra-ocular muscle movement, and elevated intraocular pressure (IOP) are seen in panophthalmitis [1].

Common causative bacteria are *Staphylococcus *species, *Klebsiella *species, *Escherichia coli*, *Pseudomonas aeruginosa*, and *Neisseria meningitidis* [2].

Panophthalmitis is largely a clinical diagnosis, but it should be supported by investigations, such as microbiological staining and cultures from the eye. Contrast-enhanced computed tomography (CECT) of the orbit will be helpful to confirm the clinical diagnosis by demonstrating scleral thickening and retrobulbar inflammation, which is seen by the presence of intraconal or extraconal fat stranding. In addition, magnetic resonance imaging (MRI) with diffusion-weighted imaging (DWI) will aid in evaluating the extent of the disease, complications, and response to treatment [3].

Here, we present a case of panophthalmitis caused by *Providencia stuartii*, an opportunistic pathogen, rarely known to cause ocular infection. *P. stuartii* causes opportunistic infections, typically in catheterised patients with urinary tract infection. To the best of our knowledge, there is no previous literature that has reported a case of endophthalmitis or panophthalmitis caused by *P. stuartii.*

## Case presentation

A 58-year-old female with underlying uncontrolled type 2 diabetes mellitus (admission random blood glucose: 18 mmol/L) presented with a painful loss of vision in the left eye for one day. She denied having a fever or any other systemic symptoms. There was no history of recent ocular trauma, ocular surgery, hospital admission, or any medical procedures. Prior to this presentation, she had good vision in both eyes. Social history revealed that she is a housewife who stays with her family at the onset of symptoms.

**Figure 1:**
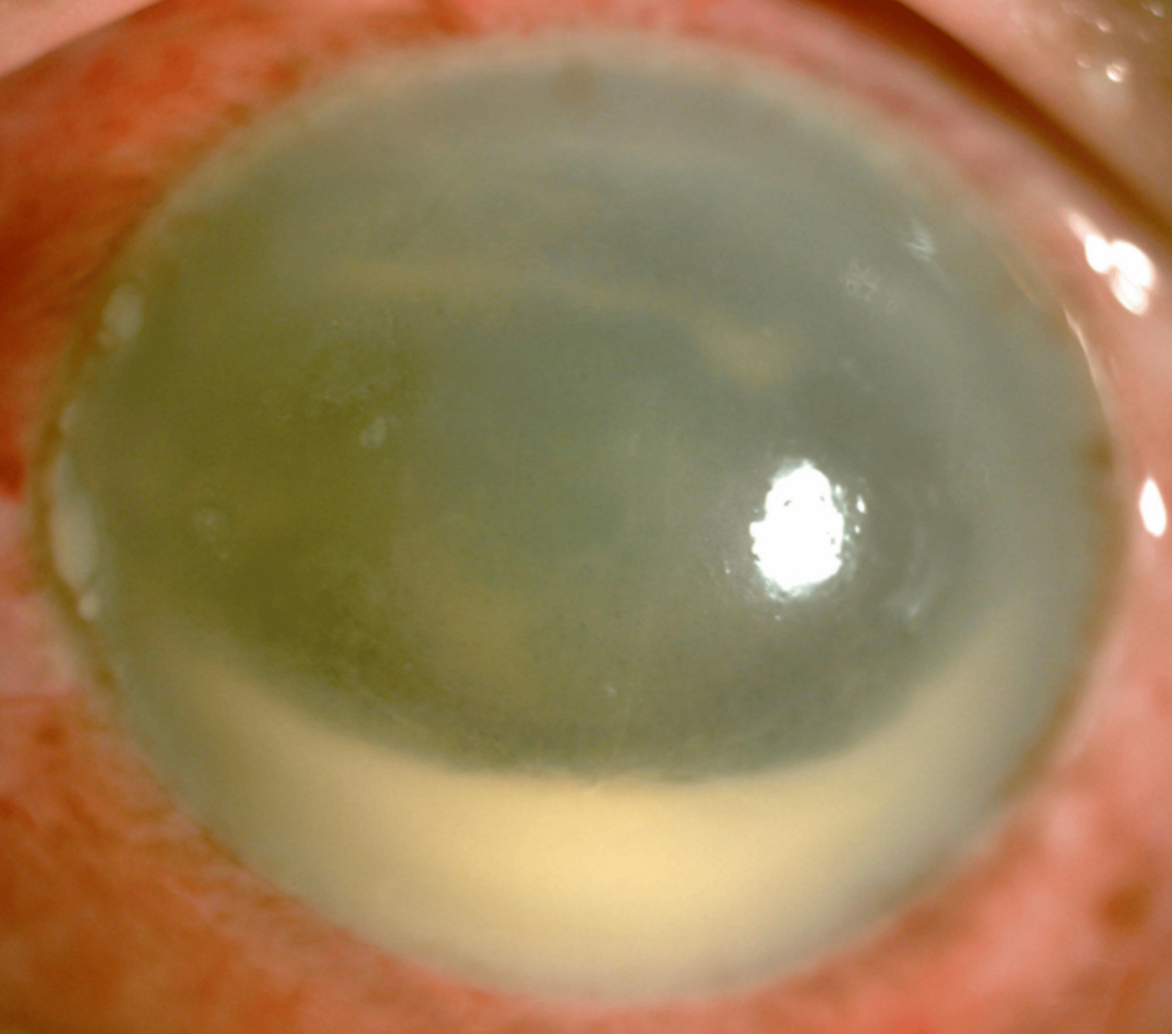
Left eye anterior segment photograph obtained at initial presentation, showing an injected conjunctiva, a hazy cornea, and a visible hypopyon.

On examination, her vital signs were stable, with a blood pressure of 125/73 mmHg, a pulse rate of 84 beats per minute, and a temperature of 37.0 °C. Ocular examination showed a visual acuity of light perception in the left eye with a positive relative afferent pupillary defect, and her right eye's aided visual acuity was 6/9. Examination of the right eye showed normal IOP of 16 mmHg, with unremarkable anterior segment and fundus findings. Left eye examination revealed limitation of extra-ocular muscle movement in all gazes, proptosis, and erythematous lid swelling. She had conjunctival injection and oedematous cornea, and IOP was elevated at 45 mmHg. The anterior chamber showed the presence of hypopyon with dense fibrin (Figure 1).

**Figure 2:**
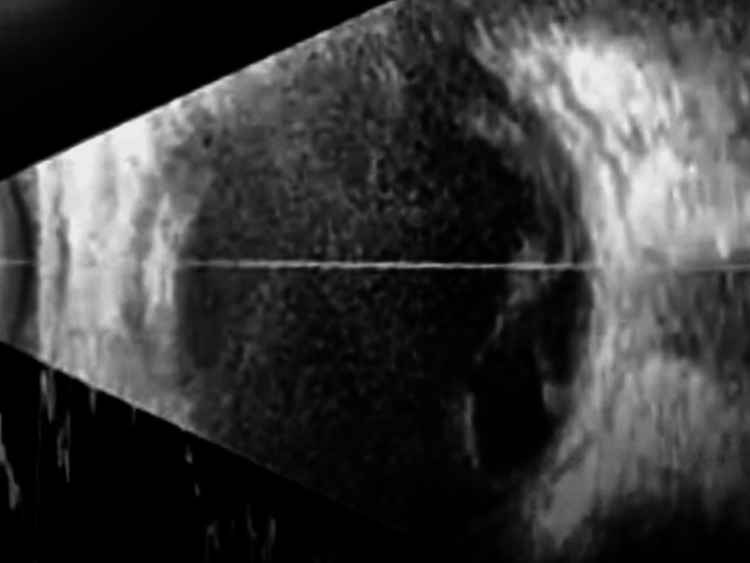
Left eye B-scan ultrasonography showing dense vitritis with loculation and fluid in the sub-Tenon space.

Laboratory investigations showed leukocytosis, with a white blood cell count of 18.38 x 10^9^/L, and her HbA1c was elevated at 10.7%. Vitreous culture from vitreous tap grew* P. stuartii*, which was sensitive to cefuroxime, cefotaxime, ceftazidime, ciprofloxacin, bactrim, and carbapenem while being resistant to gentamicin. The patient's systemic septic workup was unremarkable; blood and urine cultures showed no growth, chest X-ray was clear, echocardiography showed no valvular vegetations, and an abdominal ultrasound showed no evidence of fluid collection or occult abscesses. B-scan ultrasonography demonstrated dense vitritis, loculations, and evidence of scleritis (Figure 2). 

**Figure 3:**
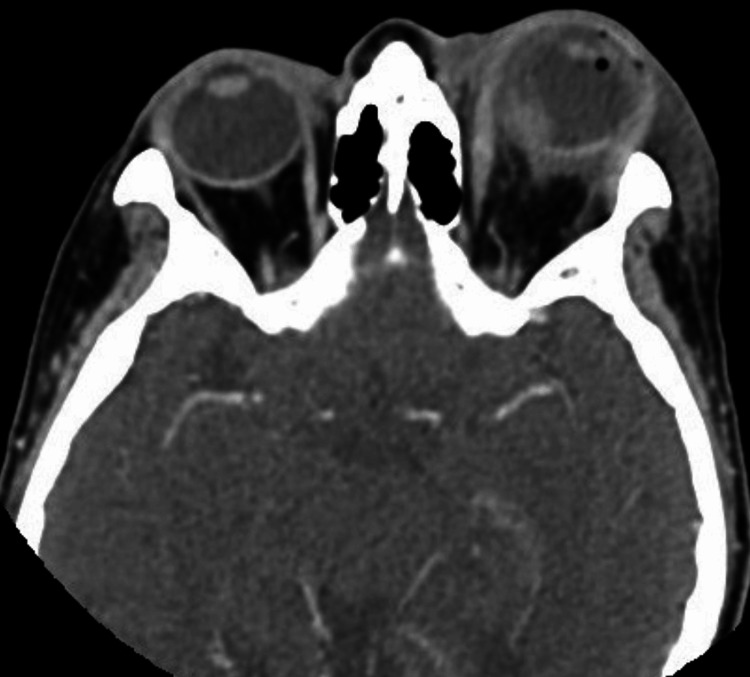
CECT orbit showing left eye proptosis, thickening of the scleral margin with choroidal enhancement, and presence of fat streakiness at the intraconal spaces.

Orbit CECT revealed left eye proptosis, circumferential thickening and enhancement of the scleral margin, choroidal enhancement, and fat stranding involving the extraconal and intraconal spaces, suggesting panophthalmitis (Figure 3).

Based on clinical and radiographic findings, a diagnosis of left eye panophthalmitis was made. Intravitreal vancomycin (2 mg in 0.1 mL) and ceftazidime (2 mg in 0.1 mL) were given every 48 hours for a total of three doses. According to the culture sensitivity, she was treated with topical ciprofloxacin and ceftazidime alongside systemic ciprofloxacin.

After three days, systemic antibiotic was changed to intravenous meropenem 2 g three times daily (TDS) due to poor clinical improvement. The patient developed total corneal melting with corneal perforation. Due to the worsening of her eye condition, left eye evisceration was done. Vitreous, uveal tissue, and conjunctival culture yielded *P. stuartii.*

Post evisceration, there was necrotic tissue at the wound, conjunctival chemosis, and eye discharge. The patient was co-managed with the oculoplastic team and treated with topical cefuroxime 5% and chloramphenicol 0.5% for a total of two weeks. On Day 7 post evisceration, chemosis and eye discharge reduced, with residual necrotic tissue at the wound.

## Discussion

*Providencia *species is a ubiquitous, Gram-negative bacterium of the Morganellaceae family. Clinically relevant species in the genus *Providencia *are *P. stuartii, P. rettgeri, P. alcalifaciens, P. rustigianii, *and *P. heimbachae*, with the most common species being *P. stuartii* [4].

*P. stuartii* is commonly found in environmental reservoirs, such as soil and water, and in animal reservoirs. It causes opportunistic infection in hospitalised patients and elderly individuals residing in nursing homes. *P. stuartii* is most commonly isolated in those who are on long-term indwelling urinary catheters with urinary tract infections [5].

*P. stuartii* has the ability to migrate from the urinary tract to other organs, such as the brain, heart, and liver [4,6]. However, it is rarely associated with ocular infections.

Lin et al. reported a case of chronic canaliculitis due to *P. stuartii* infection in an elderly female, with punctal plugs, meibomian gland dysfunction, blepharitis, conjunctivochalasis, and map-dot-fingerprint corneal dystrophy, who presented with recurrent episodes of epiphora and punctual discharge. She was treated with trimethoprim/polymyxin B according to the *P. stuartii *antibiotic susceptibility, and endoscopic dacryocystorhinostomy was performed due to residual symptoms of epiphora [5].

*P. stuartii* has also been described in the literature as a cause of conjunctivitis. Crane et al. reported a case of *P. stuartii* conjunctivitis in an elderly male living in a nursing home who was likely immunocompromised from diabetes mellitus. However, no urine culture was available. He was successfully treated with vancomycin eyedrops and oral sulfamethoxazole and trimethoprim according to culture sensitivity [7].

Koreishi et al. reported five cases of ocular infections (keratitis, dacryocystitis, conjunctivitis, and endophthalmitis) caused by *P. rettgeri.* All the patients were elderly, and three patients were immunocompromised either locally or systemically. There was a positive urinary analysis, suggesting urinary tract infection in two patients [8].

Our patient was a middle-aged female who was likely immunocompromised from longstanding uncontrolled diabetes mellitus, evidenced by a HbA1c of 10.7%. However, her urine culture and other systemic septic workup were unremarkable. This is similar to the previously reported cases, where most of their patients were also immunocompromised.

In her treatment course, intravenous ciprofloxacin was changed to intravenous meropenem, as it has been shown to have better ocular penetration and can rapidly achieve higher vitreous concentration. Available data do not support the administration of the usually used ciprofloxacin due to its insufficient concentration in the vitreous [9]. Despite timely treatment with systemic and topical antibiotics based on culture sensitivity, the ocular infection progressed rapidly, leading to corneal perforation and evisceration.

Guidone et al. found that *P. stuartii* isolates had various virulence traits, making them highly successful pathogens, and also showed high potential for antimicrobial resistance [10].

## Conclusions

*P. stuartii* infection is rare but a serious ocular infection. Despite prompt treatment, the ocular infection worsened rapidly with a poor visual outcome. This case demonstrates that *P. stuartii *can cross the boundaries of typical hospital-acquired infection to cause aggressive community-acquired infection in susceptible individuals, such as patients with uncontrolled diabetes mellitus. Despite prompt treatment with antibiotics guided by culture sensitivity, the infection progressed rapidly, requiring evisceration. This highlights an important lesson that culture sensitivity profiles for *P. stuartii* may not reliably translate to the clinical outcome in severe ocular infections. Clinicians should maintain a high index of suspicion for rapid disease progression with this pathogen and consider early escalation of antibiotics when first-line treatment shows a poor clinical response.
